# Quantification of Skeletal Muscle Perfusion in Feet and Lower Legs of Patients with T2DM and Diabetic Foot Ulcers Using [^15^O]H_2_O PET

**DOI:** 10.3390/jcm14217704

**Published:** 2025-10-30

**Authors:** Nana Louise Christensen, Lars Poulsen Tolbod, Jens Sörensen, Kirsten Bouchelouche, Michael Alle Madsen, Christian Selmer Buhl

**Affiliations:** 1 Department of Nuclear Medicine, Aarhus University Hospital, 8200 Aarhus, Denmark; 2 Steno Diabetes Center, Aarhus University Hospital, 8200 Aarhus, Denmark; chrisbuh@rm.dk; 3 Department of Clinical Medicine, Aarhus University, 8200 Aarhus, Denmark

**Keywords:** diabetes, diabetic foot ulcer, positron emission tomography, perfusion

## Abstract

**Background/Objectives**: Type 2 Diabetes Mellitus (T2DM) represents a significant global health burden, frequently leading to severe complications such as peripheral neuropathy and both micro- and macrovascular dysfunctions. These complications are integral to the pathology of diabetic foot ulcers (DFUs) and are associated with an elevated risk of lower limb amputations. This study investigated lower extremity skeletal muscle perfusion in patients with T2DM and DFUs using [^15^O]H_2_O PET imaging, in comparison to healthy controls. **Methods**: A total of 10 healthy controls and 26 patients with T2DM and DFUs were enrolled. Resting skeletal muscle perfusion was quantified using [^15^O]H_2_O PET. Regional perfusion was assessed in multiple lower leg and foot muscle groups. Distal blood pressure was measured, and foot/leg perfusion ratios were calculated. **Results**: Patients with T2DM and DFUs exhibited a 58% higher median global foot resting perfusion compared to healthy controls. At the individual muscle level, median perfusion in the flexor hallucis brevis was elevated by up to 210% in the ulcerated foot compared to controls. No significant differences in perfusion were observed in the non-ulcerated foot. The foot/leg perfusion ratio was up to 58% higher in the ulcerated limb of T2DM patients compared to the controls. **Conclusions**: This study demonstrates localized alterations in skeletal muscle perfusion in patients with T2DM and DFUs, characterized by elevated resting foot perfusion in muscles adjacent to ulcerations. Understanding these perfusion dynamics may contribute to refined DFU management strategies. However, further research is needed to validate the clinical utility of [^15^O]H_2_O PET imaging in guiding interventions and predicting treatment outcomes for DFUs.

## 1. Introduction

Diabetes Mellitus (DM) represents a global health burden with substantial implications for both individuals and healthcare systems. Affecting an estimated 463 million individuals, DM markedly diminishes quality of life and ranks among the ten leading causes of global mortality [1,2,3].

Among its numerous complications, diabetic foot ulcers (DFUs) represent a particularly concerning issue, associated with increased morbidity and mortality rate, a heightened risk of lower limb amputation, and considerable healthcare costs [4]. Both micro- and macrovascular dysfunction, including impaired perfusion, play a fundamental role in the pathogenesis of DFUs in patients with Type 2 Diabetes Mellitus (T2DM) [5]. The lifetime incidence of DFUs among individuals with diabetes ranges from 15% to 25% [6], with approximately one in five ulcers ultimately requiring amputation [7]. At this advanced stage, the 5-year mortality rate is comparable to that of many malignancies, underscoring the severity of this clinical condition [5,8]. Precise initial diagnostics are therefore critical to mitigate unnecessary readmissions and subsequent amputations [9].

The diagnosis and management of DFU conventionally involve the evaluation of distal blood pressure via ankle-to-brachial (ABI) and toe-to-brachial (TBI) ratios. However, these measurements can be complicated by factors such as edema, ulcers, pain, and tremor in patients [10]. While laser Doppler technology has shown promise in assessing distal blood pressure in individuals with lower limb perfusion deficits and ulcers [11], its results remain susceptible to influences like motion, temperature fluctuations, and inter-operator variability [12]. Furthermore, in diabetic patients, ankle pressure measurements may be falsely elevated due to media sclerosis and increased vessel wall rigidity, rendering toe pressure a more reliable indicator [13], despite its limitations in differentiating perfusion among specific muscle groups [14].

Various imaging modalities, including magnetic resonance imaging (MRI) [15,16,17,18,19], single-photon emission computed tomography (SPECT) [20,21,22,23,24,25], and positron emission tomography (PET) [14,26,27] have been proposed for evaluating lower extremity perfusion. PET, a widely employed non-invasive method for quantifying tissue blood flow, has demonstrated encouraging results in the assessment of lower limb perfusion [14,26,28,29,30,31,32].

This study aimed to assess lower extremity resting skeletal muscle perfusion in patients with T2DM and DFUs using [^15^O]H_2_O PET imaging and to compare these findings with those obtained from healthy controls, thereby investigating potential differences between the two groups.

## 2. Materials and Methods

### 2.1. Study Population

Initially, ten healthy individuals served as controls. Exclusion criteria were applied to ensure a homogenous control group, including a history of vascular surgery, leg pain (including rest pain), current tobacco use (including nicotine products), age outside the range of 40–90 years, pregnancy, claustrophobia, severe obesity, or prior chemotherapy. Subsequently, 26 patients diagnosed with T2DM and DFUs were enrolled. Exclusion criteria for patients mirrored those of the control group, with the addition of severe lung or heart disease (e.g., heart failure with ejection fraction < 20%, NYHA class IV) or malignancy. Inclusion and exclusion parameters were established to define a specific demographic and clinical profile, facilitating a focused investigation of the research objectives. The patients had the best possible offloading strategy, none had prior amputations, and all had DFUs at the time of examination. The study was conducted in accordance with the Declaration of Helsinki and was approved by the Central Denmark Region Committees on Health Research Ethics, with informed consent obtained from all participants.

### 2.2. [^15^O]H_2_O PET Imaging of Skeletal Muscle Perfusion

Participants underwent [^15^O]H_2_O PET scans following a standardized protocol, including a minimum 2 h fasting period and 24 h abstinence from caffeine and nicotine. Scans were performed using a Siemens Vision PET/CT system (Siemens Healthineers, Knoxville, TN, USA) with a 26 cm axial field-of-view. The protocol comprised a low-dose CT scan of the feet, lower legs, and heart for attenuation correction, followed by 6 min dynamic list mode PET acquisitions of the same regions. A 400 MBq intravenous bolus of [^15^O]H_2_O was administered at the start of each PET acquisition using a MedRad CT Injection System (1.0 mL/s, MEDRAD Inc., Warrendale, PA, USA). PET images were reconstructed to 2.75 × 2.75 × 3 mm^3^ voxels using a time-of-flight ordered-subset expectation maximization algorithm (TrueX, 4 iterations, 5 subsets), with all necessary corrections applied.

### 2.3. Distal Blood Pressure Measurements

Distal systolic pressures were obtained using a PeriFlux 5000 Laser Doppler System (Perimed AB, Jarfalla, Sweden) in a temperature-controlled environment. Participants rested supine for approximately 30 min with warmed feet. Toe systolic pressure was measured with photoplethysmographic laser-Doppler probes placed on the pulp of the first toes and pneumatic cuffs positioned at the base of the toes. The system automatically corrected for hydrostatic height differences between the heart and the measurement site when entered in centimeters. Ankle systolic pressure was measured using pneumatic cuffs placed just above the malleoli and a Doppler probe over the dorsalis pedis or posterior tibial artery. For both sites, the occlusion pressure was set to approximately 200 mmHg and gradually released until flow reappeared. Brachial systolic pressure was measured in the contralateral arm using an automated sphygmomanometer. The ankle-brachial index (ABI) was calculated as ankle systolic pressure divided by brachial systolic pressure, and the toe-brachial index (TBI) as toe systolic pressure divided by brachial systolic pressure. Measurements followed institutional standard operating procedures for peripheral circulation testing (Department of Nuclear Medicine, Aarhus University Hospital, Aarhus, Denmark). The procedure includes pre-warming of the feet, appropriate cuff sizing, and repeated measurements allowing <10 mmHg variation between readings.

### 2.4. Image Analysis

Image analysis was performed using PMOD software (version 4.006, PMOD Technologies GmbH, Zurich, Switzerland). Manual delineation of volumes of interest (VOIs) within skeletal muscles of the feet and lower legs was conducted using both CT images and summed early PET frames (1–10) to exclude major arterial vessels. Analyzed muscle groups included the abductor digiti minimi (ADM), abductor hallucis (AH), flexor digitorum brevis (FDB), and flexor hallucis brevis (FHB) in the feet, and gastrocnemius (GAS), peroneus longus (PL), soleus (SOL), and tibialis anterior (TA) in the lower legs. Tissue perfusion was quantified via the autoradiographic method (ARG) [30] using aQuant Research (MedTrace Pharma A/S, Hørsholm, Denmark) from extracted time-activity curves (TACs). Detailed analytical procedures, including blood input function derivation, arterial TAC extraction, delay and dispersion assessment, and parametric image generation, have been previously described [33].

### 2.5. Comparative Analyses and Definitions

The primary objective was to assess resting perfusion levels in the feet and lower legs within the context of two distinct groups: healthy controls and patients with T2DM and DFUs. To achieve this, we conducted comparisons across various muscle groups for controls and two specific subsets of patients: perfusion values obtained from the ulcerated and the non-ulcerated lower extremity. For controls, mean perfusion values were calculated for muscle groups in both lower extremities. When comparing global perfusion values, we used the mean perfusion across four muscle groups in the lower legs and feet, respectively. These estimations were then used to calculate foot/leg perfusion ratios for both groups. The patients were divided into two groups based on their TBI, using the median TBI for the patients as a cut-off (TBI > 0.56 or TBI ≤ 0.56). This cutoff divides the patient population into two groups: those exhibiting more pronounced vascular impairment (TBI ≤ 0.56) and those with milder impairment (TBI > 0.56). This enables a clinically relevant comparison of perfusion patterns, directly addressing the critical role of vascular compromise severity in the development and management of DFUs. Furthermore, we explored the differences in resting perfusion for specific muscle groups in the feet of patients: (1) the muscle closest to the DFU, (2) the corresponding muscle in the non-ulcerated foot, (3) a muscle away from the ulcer in the ulcerated foot, and 4) the corresponding muscle in the non-ulcerated foot.

The comparison in the feet was dependent on the location of the DFU in the ulcerated foot. If the muscle closest to the DFU was identified as FHB, AH, or FDB, then ADM was considered the away muscle for comparison. Conversely, if ADM was the muscle closest to the DFU, FHB was designated as the away muscle.

### 2.6. Statistical Methods

Data distribution was assessed using histograms and the Shapiro–Wilk test. Differences between groups were evaluated using the Wilcoxon signed-rank test or Mann–Whitney U test for non-normally distributed data, and Student’s *t*-test for normally distributed data. Spearman’s correlation was used to assess associations. Pairwise comparisons were conducted without correction for multiple comparisons, as the analyses were exploratory in nature. Statistical significance was set at *p* < 0.05, and all analyses were performed using Stata 17.0 (StataCorp, College Station, TX, USA).

## 3. Results

### 3.1. Subject Demographics

The demographic and clinical characteristics of the study cohort are summarized in Table 1. The cohort comprised 26 patients, with a predominant male representation (92%). All patients exhibited neuropathy, 35% presented with peripheral ischemia, indicated by a TBI < 0.7 [34]. Healthy controls were, on average, nine years younger compared to the patients, with a comparable BMI (29.39 kg/m^2^ vs. 29.82 kg/m^2^, *p* = 0.83). The control group was 70% male. Healthy controls were free of known cardiovascular disease and were not receiving antihypertensive medication. Blood pressure values reflect single-day measurements after rest, and occasional elevated systolic readings do not indicate established hypertension.

### 3.2. Lower Leg Perfusion in Patients with T2DM and Healthy Controls

**Figure 1:**
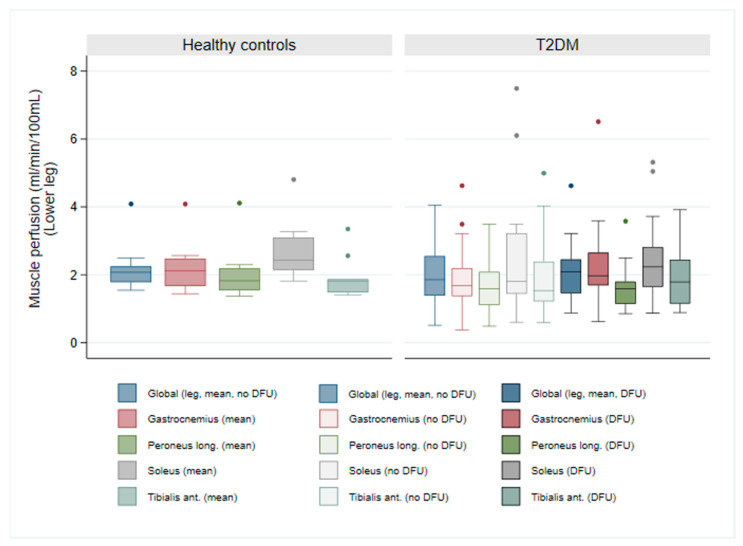
Comparison of resting skeletal muscle perfusion in lower legs between healthy controls and patients with T2DM and DFUs, in the non-ulcerated leg (T2DM no DFU) and the ulcerated leg (T2DM DFU). The box plot illustrates the perfusion values of four specific muscle groups in the lower legs and global leg perfusion (leg, mean, no DFU and leg, mean, DFU).

Resting skeletal muscle perfusion in the lower legs revealed no significant disparities between patients and controls, irrespective of ulceration status. Median perfusion values in the ulcerated leg ranged from 1.59 to 2.23 mL/min/100 mL, closely mirroring those observed in the non-ulcerated leg (1.54 to 1.85 mL/min/100 mL—see Table 2). Although not statistically significant, a trend towards slightly elevated perfusion was noted in the ulcerated leg compared to the non-ulcerated leg within the T2DM group, particularly within the gastrocnemius muscle (1.97 vs. 1.68 mL/min/100 mL, *p* = 0.08). As illustrated in Figure 1, global leg resting perfusion exhibited no differences between healthy controls and patients with T2DM, in either the ulcerated leg (*p* = 0.66) or the non-ulcerated leg (*p* = 0.36).

### 3.3. Foot Perfusion of Patients with T2DM and DFUs Compared to Healthy Controls

Table 2 presents the median resting perfusion values for different foot muscle groups in controls and patients. Resting perfusion ranged from 0.45 to 3.20 mL/min/100 mL in controls, while patients exhibited values from 0.23 to 5.99 mL/min/100 mL in the non-ulcerated foot and 0.50 to 9.51 mL/min/100 mL in the ulcerated foot. Significantly higher median resting perfusion was observed in three muscle groups in the ulcerated foot compared to controls: AH (2.04 vs. 1.06 mL/min/100 mL, *p* < 0.001), FDB (1.87 vs. 1.28 mL/min/100 mL, *p* = 0.002) and FHB (2.91 vs. 0.94 mL/min/100 mL, *p* < 0.001). However, no statistically significant difference was found in ADM (*p* = 0.05). Table 2 also demonstrates significantly lower toe pressure and TBI in the ulcerated foot of patients compared to controls (toe pressure: 73.2 vs. 110.1 mmHg, *p* < 0.001; TBI: 0.59 vs. 0.92, *p* < 0.001), and between the ulcerated and non-ulcerated foot within the T2DM cohort (toe pressure: 73.2 vs. 83.4 mmHg, *p* = 0.04; TBI: 0.59 vs. 0.67, *p* = 0.03).

**Figure 2:**
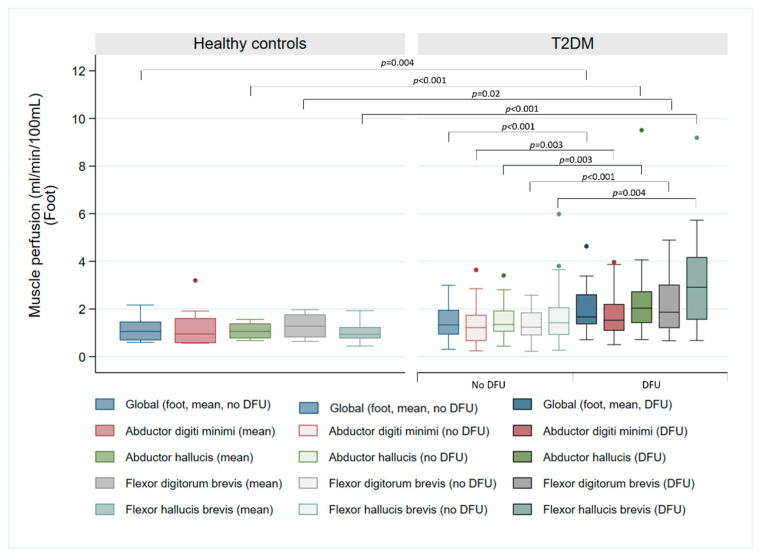
Comparison of resting skeletal muscle perfusion in the feet between healthy controls and patients with T2DM and DFUs, in the non-ulcerated foot (T2DM no DFU) and the ulcerated foot (T2DM DFU). The box plot illustrates the perfusion values of four specific muscle groups in the feet and global foot perfusion (foot, mean, no DFU, and foot, mean, DFU).

As illustrated in Figure 2, global resting perfusion differed significantly between controls and the ulcerated foot of patients (*p* < 0.005), but not in the non-ulcerated foot (*p* = 0.22).

Among the patients, the FHB muscle was the most commonly affected site for DFUs, accounting for 73% of cases. There was a significant difference in FHB perfusion between controls and the muscle closest to the DFU (0.94 vs. 3.02 mL/min/100 mL, *p* < 0.001), and when compared to the corresponding muscle in the contralateral foot (0.94 vs. 1.31 mL/min/100 mL, *p* = 0.02) (Figure S1).

### 3.4. Perfusion Differences Among Muscle Groups in Patients with T2DM and DFU

Analysis of individual muscle groups (Figure S2) revealed significantly elevated resting perfusion in the muscle closest to the ulcer compared to: (1) A distant muscle within the same ulcerated foot (3.02 vs. 1.53 mL/min/100 mL, *p* < 0.001), (2) The corresponding muscle in the non-ulcerated foot (3.02 vs. 1.31 mL/min/100 mL, *p* = 0.001) and (3) A distant muscle in the non-ulcerated foot (3.02 vs. 1.41 mL/min/100 mL, *p* < 0.001).

### 3.5. Foot/Leg Perfusion Ratio and Its Relationship with TBI in Patients with T2DM

**Figure 3:**
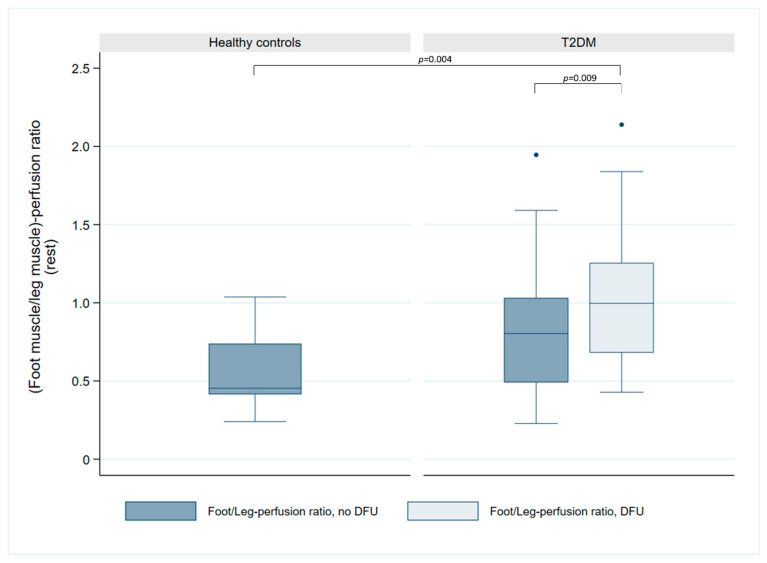
Comparison of foot/leg perfusion ratio in healthy controls and patients with T2DM and DFUs. The box plot illustrates the perfusion ratio in the non-ulcerated foot/leg (blue) compared to the ulcerated foot/leg (light blue). Significance levels are indicated for the observed significant differences between groups.

Examination of the foot/leg perfusion ratio (Figure 3) demonstrated a significant difference between controls and patients with T2DM in the ulcerated leg (0.45 vs. 1.00, *p* = 0.004), with a trend towards difference in the non-ulcerated leg (0.45 vs. 0.80, *p* = 0.057). Within the T2DM patients, perfusion ratio was significantly higher in the ulcerated vs. the non-ulcerated leg (1.00 vs. 0.80, *p* = 0.009).

**Figure 4:**
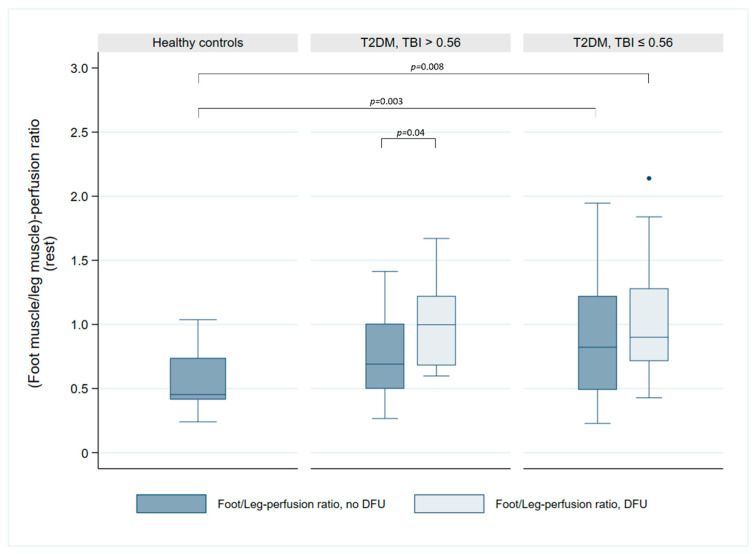
Comparison of foot/leg perfusion ratio in healthy controls and patients with T2DM and DFUs in relation to their TBI. The patients are divided into two subgroups: TBI > 0.56 and TBI ≤ 0.56. The box plot illustrates the perfusion ratio in the non-ulcerated foot/leg (blue) compared to the ulcerated foot/leg (light blue). Significance levels are indicated for the observed significant differences between groups.

The analysis of the foot/leg perfusion ratio and hemodynamic parameters (Figure 4) revealed that, in patients with a TBI ≤ 0.56, the perfusion ratio was significantly reduced in both the non-ulcerated (0.82 vs. 0.45, *p* = 0.003) and the ulcerated leg (0.90 vs. 0.45, *p* = 0.008) compared to healthy controls. However, no significant differences were observed between groups for either the non-ulcerated leg (0.45 vs. 0.69, *p* = 0.06) or the ulcerated leg (0.45 vs. 1.00, *p* = 0.14). A significant inter-limb difference was evident within the TBI > 0.56 subgroup (0.69 vs. 1.00, *p* = 0.04) but absent in the TBI ≤ 0.56 subgroup (0.82 vs. 0.90, *p* = 0.1). Furthermore, the foot/leg perfusion ratio did not differ significantly between TBI subgroups for either the non-ulcerated (0.69 vs. 0.82, *p* = 0.94) or ulcerated legs (1.00 vs. 0.90, *p* = 0.49). Spearman correlation analysis revealed no association between TBI and the foot/leg perfusion ratio (r = −0.04, *p* = 0.84 in the non-ulcerated leg; r = 0.16, *p* = 0.45 in the ulcerated leg).

## 4. Discussion

This study investigated variations in resting perfusion of lower legs and feet between patients with T2DM and DFUs and healthy controls, aiming to elucidate vascular dynamics associated with DFU pathogenesis. While only minimal differences were detected in resting perfusion of lower legs, our results align with a prior MRI study, which similarly found no significant distinction between healthy controls and patients with T2DM with and without DFU [16]. Similarly, differences between the DFU-affected leg and the contralateral leg in patients with T2DM were marginal.

Patients with T2DM and DFUs exhibited elevated resting perfusion values in the ulcerated foot across all assessed muscle groups, potentially representing a compensatory vasodynamic response to the ulceration driven by inflammatory mediators and vasoactive substances like serotonin, bradykinin, prostaglandins, and histamine. These substances increase endothelial permeability and stimulate localized perfusion around the wound, promoting wound healing processes [35], and likely contributing to the observed elevation in resting perfusion values. Our investigation of foot perfusion revealed intriguing patterns. Patients with T2DM and DFUs displayed significantly higher median resting perfusion in the ulcerated foot compared to healthy controls in the abductor hallucis (AH), flexor digitorum brevis (FDB), and flexor hallucis brevis (FHB) muscle groups. Furthermore, while perfusion generally trended to be higher in the non-ulcerated foot of patients compared to controls across all muscle groups, these differences did not reach statistical significance. Similar patterns were found when comparing the foot/leg perfusion ratio between groups, with the ratio generally higher in the patients in both the ulcerated and the non-ulcerated limbs. These results suggest that changes in foot perfusion may be more pronounced in muscle groups directly affected by DFU.

Outliers within the control group were attributable to a single individual and likely resulted from physical exercise performed earlier on the scan day prior to scanning. In the patient group, outliers were linked to patient motion during scanning, increasing blood signal within the VOIs, leading to increased perfusion values.

### 4.1. Toe Pressure/TBI and PET Perfusion Values

The observed elevation in resting perfusion within feet and skeletal muscles affected by DFUs, combined with reduced toe pressure and TBI, may be explained by factors linked to the pathophysiology of diabetic vascular complications and wound healing processes. The dysfunction of endothelial cells observed in DM can disrupt their ability to properly respond to changes in blood vessel size, causing imbalances in dilation and constriction. Consequently, this can lead to regional variations in perfusion, where certain areas exhibit elevated perfusion values despite the overall compromised circulation [36]. Chronic hyperglycemia directly affects endothelial cells, modulating vessel diameter and stimulating excessive nitric oxide (NO) production—a potent vasodilator critical for blood flow regulation. However, this NO-mediated vasodilation can manifest as inappropriate vasodilation, leading to augmented blood flow [37]. In the context of the relationship between the foot/leg perfusion ratio and TBI among the patients, we did not find a difference between the perfusion ratios in the TBI subgroups.

### 4.2. Increased Perfusion in DFU-Affected Foot Compared to Contralateral Foot

Our results demonstrate significant differences in resting foot perfusion between the ulcerated and non-ulcerated foot in patients with T2DM and DFUs. This indicates that DFUs alter perfusion dynamics, likely through local vasodilation and blood flow redistribution to support wound healing. Such localized changes may also have systemic effects, influencing perfusion in the non-ulcerated foot. Factors such as glycemic control, muscle atrophy, and systemic effects in diabetes could contribute to this observation. Increased perfusion in non-ulcerated foot muscles may reflect compensatory mechanisms against sarcopenia or better microvascular function associated with glycemic control. Stratton et al. [38] similarly reported that improved control is linked to less severe microvascular dysfunction, consistent with our findings. Diabetes-related factors such as inflammation, oxidative stress, endothelial dysfunction, and neuropathy further add to the variability in perfusion responses. At the muscle level, perfusion revealed distinct differences within patients with T2DM when comparing the foot muscle groups closest to the DFU with those in the corresponding muscle group in the contralateral foot. This finding suggests localized perfusion dynamics within the tissues surrounding the DFU, further emphasizing the role of perfusion in diabetic foot complications. Notably, a discernible difference existed between the foot muscle closest to the DFU and more distal muscles within the same foot, suggesting that the ulcer itself modulates perfusion in adjacent tissues, likely due to localized inflammation and hemodynamic alterations. The observed disparity between the muscle closest to the DFU and its counterparts in the non-ulcerated foot indicates that the perfusion alterations are specifically linked to the presence of the ulcer and not solely related to the muscle itself. Our findings align with previous research investigating foot perfusion in patients with T2DM and DFUs. An MRI study utilizing arterial spin labeling demonstrated hyperemia in the wound bed and surrounding tissue, exhibiting a 50% increase in perfusion compared to more distant tissue [39]. Similarly, a dynamic 4DCT study reported significantly elevated perfusion in affected tissue compared to unaffected tissue in all three participants [40].

**Figure 5:**
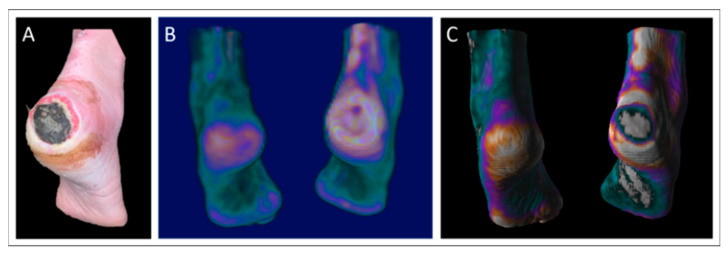
Clinical case from the patient cohort: A 56-year-old male with a severe non-healing ulcer (80 × 80 × 2 mm) on the right heel (**A**), along with sagittal parametric maximum intensity projection PET images in both non-fused (**B**) and fused with CT (**C**) formats. Resting perfusion values for this specific patient ranged from 1.33 to 2.07 mL/min/100 mL within the muscle groups of the ulcerated foot. Color scale represents quantitative perfusion values, with white indicating the highest perfusion, purple representing intermediate levels, and turquoise indicating the lowest perfusion.

An illustrative case from our cohort was a 56-year-old male with neuropathy and a large heel DFU (80 × 80 × 2 mm, Figure 5A). The ulcer showed necrosis, granulation tissue, localized redness, warmth, and edema with moderate exudation. PET and PET/CT images (Figure 5B,C) revealed increased perfusion surrounding the ulcer and the absence of perfusion in necrotic regions. Perfusion values ranged from 1.33 to 2.07 mL/min/100 mL in affected foot muscles.

### 4.3. Future Perspectives

The utilization of [^15^O]H_2_O PET for assessing tissue perfusion in T2DM patients with DFU holds potential for future applications in treatment monitoring. This method could serve as a predictor of wound healing and offer guidance in revascularization decisions or amputation level determination. However, further studies are needed to establish the robustness and clinical applicability of [^15^O]H_2_O PET in diverse patient populations and treatment contexts. Crucially, studies should evaluate its sensitivity to therapeutic interventions, its prognostic value for long-term outcomes, and its comparative efficacy relative to established perfusion assessment methodologies to fully elucidate its clinical potential. Findings from studies like that of Chou et al., demonstrating substantial post-revascularization perfusion improvements with SPECT, further underscore the importance of precise perfusion assessment in guiding effective interventions for vascular complications in T2DM [24].

Moreover, integrating the use of [^18^F]FDG PET/CT could enhance diagnostic capabilities, particularly in identifying inflammatory complications related to DFUs, such as osteomyelitis, acute Charcot foot, and soft tissue infections. Recent guidelines from the European Association of Nuclear Medicine (EANM) recommend [^18^F]FDG PET/CT for these purposes. Such investigations could determine whether elevated perfusion is due to inflammatory healing responses or infectious processes [41].

### 4.4. Limitations

All reported perfusion values relate to resting conditions, and we did not explore the potential for perfusion increase during post-exercise or pharmacological stress testing. Investigating these hemodynamics could provide further insights into the vascular reactivity and blood flow reserve capacity in diabetic foot ulceration.

During the scans, small movements in the feet occurred, which we were unable to correct. These movements have the potential to introduce measurement errors, particularly in VOI-based analysis. Motion-induced signals from blood vessels within the VOI could potentially lead to falsely elevated perfusion values.

The lack of statistical significance in our study may be attributed to the inherent complexity of perfusion regulation in diabetic individuals, the sample size, or inter-participant variability in disease severity. However, our prior test–retest repeatability analyses suggest that the sample size was adequate to detect clinically relevant changes in perfusion [33]. These analyses indicated that sample sizes of 10, 5, and 4 would be sufficient to detect 25%, 50%, and 75% increase in lower leg perfusion, respectively, with a 95% power. For the feet, the required sample sizes were 18, 7, and 5. Given that our cohort involved 26 patients with T2DM, we were adequately powered to detect changes in lower leg perfusion, but potentially underpowered to detect subtle differences in foot perfusion.

## 5. Conclusions

This study investigated the relationship between T2DM, DFUs, and resting skeletal muscle perfusion using [^15^O]H_2_O PET imaging. Although no significant differences were observed in resting perfusion of the lower legs, notable disparities in resting foot perfusion were detected in patients with T2DM and DFUs compared to healthy controls, suggesting localized vasodynamic alterations likely attributable to the inflammatory response associated with wound healing. Patients with T2DM and DFUs displayed elevated resting foot perfusion within specific muscle groups, potentially representing a compensatory response to ulceration-induced changes. These alterations were more pronounced in muscle groups located close to the DFUs, underscoring the heterogeneous impact of DFUs on regional muscle perfusion. Understanding these perfusion dynamics may facilitate the development of refined management strategies for DFUs, improving healing trajectories and patient outcomes.

## Figures and Tables

**Table 1:** Subject demographics.

	Controls (*n* = 10)	Patients (*n* = 26)
Age (years)	53.00 ± 7.75	62.07 ± 8.79
Male/female	7/3	24/2
Height (m)	1.78 ± 0.07	1.82 ± 0.06
Weight (kg)	92.70 ± 24.40	98.85 ± 14.12
Body Mass Index (kg/m^2^)	29.39 ± 7.67	29.82 ± 4.11
Systolic blood pressure (mmHg) *	131.10 ± 26.67	124.73 ± 17.18
Diastolic blood pressure (mmHg) *	76.60 ± 11.48	73.23 ± 9.71
Heart rate (bpm) *	59.30 ± 9.45	74.65 ± 12.13
Ankle pressure (mmHg)	154.65 ± 19.52	119.37 ± 34.75
Toe pressure (mmHg)	110.00 ± 19.10	69.12 ± 31.51
Ankle-Brachial Index	1.29 ± 0.08	1.00 ± 0.27
Toe-Brachial Index	0.92 ± 0.11	0.56 ± 0.24
HbA1c (mmol/mol)	37.50 ± 3.56	59.88 ± 17.21
Cholesterol (mmol/L)	5.17 ± 1.12	3.58 ± 0.88
HDL Cholesterol (mmol/L)	1.54 ± 0.56	1.05 ± 0.34
LDL Cholesterol (mmol/L)	3.00 ± 1.00	1.54 ± 0.70
eGFR/1.73 m^2^ (CKD-EPI) (mL/min)	>90 [IQR 90–90]	74.50 [IQR 64–90]
Hemoglobin (mmol/L)	8.75 [IQR 8.10–9.30]	8.65 [IQR 7.90–9.30]
Urine ACR (mg/g)	6.00 [IQR 3.00–7.00]	20.00 [IQR 9.00–62.00]
Proportion of micro/macro albuminuria	0/0	13/13
Peripheral ischemia **	0 (0%)	9 (35%)
Neuropathy	0 (0%)	26 (100%)
Diabetes duration (years)	-	13.5 ± 8.20
Biothesiometry left (volts)	-	50 [IQR 45–50]
Biothesiometry right (volts)	-	50 [IQR 41–50]
Baseline ulcer surface area (cm^2^)	-	1.88 ± 3.47
Ulcer location (left/right)	-	14/12

Continuous variables are presented as mean ± SD or median [interquartile range, IQR], as appropriate. Categorical variables are presented as numbers. * Measured after at least 5 min of resting in supine position. ** Toe-Brachial Index < 0.7.

**Table 2:** Foot and lower leg perfusion values in healthy controls and patients with T2DM and DFUs, alongside corresponding toe pressure and TBI.

	Controls	Patients	
		Non-Ulcerated Foot	Ulcerated Foot
Feet			
Abductor digiti minimi	0.95 [0.43–1.47]	1.22 [0.67–1.77] #	1.53 [0.97–2.09]
Abductor hallucis	1.06 [0.75–1.37]	1.35 [0.91–1.79] #	2.04 [1.38–2.70] *
Flexor digitorum brevis	1.28 [0.80–1.76]	1.24 [0.76–1.72] #	1.87 [0.96–2.78] *
Flexor hallucis brevis	0.94 [0.70–1.18]	1.42 [0.84–2.00] #	2.91 [1.60–4.22] *
Foot perfusion	1.06 [0.67–1.46]	1.33 [0.82–1.85] #	1.67 [1.05–2.30] *
Lower legs			
Gastrocnemius	2.12 [1.72–2.52]	1.68 [1.27–2.09]	1.97 [1.49–2.45]
Peroneus longus	1.82 [1.49–2.15]	1.59 [1.10–2.08]	1.59 [1.26–1.92]
Soleus	2.42 [1.94–2.90]	1.81 [0.92–2.70]	2.23 [1.65–2.82]
Tibialis anterior	1.82 [1.63–2.30]	1.54 [0.96–2.12]	1.79 [1.15–2.43]
Lower leg perfusion	2.07 [1.84–2.30]	1.85 [1.01–2.69]	2.08 [1.58–2.57]
Hemodynamic measurements			
Toe pressure	110 ± 19.10	82.5 ± 31.26 *#	73.2 ± 30.90 *
TBI	0.92 ± 0.11	0.67 ± 0.24 *#	0.59 ± 0.23 *

Median resting perfusion values (mL/min/100 mL, [IQR]) observed in different muscle groups of the feet and lower legs among healthy controls, and among patients with T2DM and DFUs. The patients are divided into ulcerated and non-ulcerated feet. Additionally, toe pressure (mmHg) and toe-brachial index (TBI) are presented for each group. Statistical significance is denoted as * when compared to healthy controls, and # when compared to the ulcerated foot (*p* < 0.05).

## Data Availability

Data are available from the corresponding author upon reasonable request. Participant privacy precludes public data sharing.

## Supplementary Materials

The following supporting information can be downloaded at: https://www.mdpi.com/article/10.3390/jcm14217704/s1. Figure S1: Comparison of muscle perfusion in controls (FHB muscle) and T2DM patients in the muscle closest to the DFU and the same muscle in the contralateral foot.; Figure S2: Comparison of skeletal muscle perfusion in the feet of T2DM patients.

## Abbreviations

The following abbreviations are used in this manuscript:

ABI	Ankle Brachial Index
ADM	Abductor Digiti Minimi
AH	Abductor Hallucis
ARG	Autoradiographic Method
BMI	Body Mass Index
BPM	Beats per Minute
CKD-EPI	Chronic Kidney Disease Epidemiology Collaboration
CT	Computed Tomography
DFU	Diabetic Foot Ulcer
eGFR	Estimated Glomerular Filtration Rate
EANM	European Association of Nuclear Medicine
FHB	Flexor Hallucis Brevis
FDB	Flexor Digitorum Brevis
GAS	Gastrocnemius
HbA1c	Glycated Hemoglobin
IQR	Interquartile Range
MBq	Megabecquerel
MRI	Magnetic Resonance Imaging
NO	Nitric Oxide
NYHA	New York Heart Association
PET	Positron Emission Tomography
PET/CT	Positron Emission Tomography/Computed Tomography
PL	Peroneus Longus
SD	Standard Deviation
SPECT	Single Photon Emission Computed Tomography
SOL	Soleus
T2DM	Type 2 Diabetes Mellitus
TA	Tibialis Anterior
TAC	Time Activity Curve
TBI	Toe Brachial Index
[^15^O]H_2_O	Oxygen-15 Labeled Water
[^18^F]FDG	Fluorine-18 Fluorodeoxyglucose
