# Supplementary material for: Quantification of Skeletal Muscle Perfusion in Feet and Lower Legs of Patients with T2DM and Diabetic Foot Ulcers Using [15O]H2O PET
Source: J Clin Med. 2025 Oct 30;14(21):7704. doi: 10.3390/jcm14217704 (PMC12608334; doi:10.3390/jcm14217704)
Supplement: Supplementary file 1 [file jcm-14-07704-s001.zip › jcm-3958349-supplementary.pdf]

Supplementary material

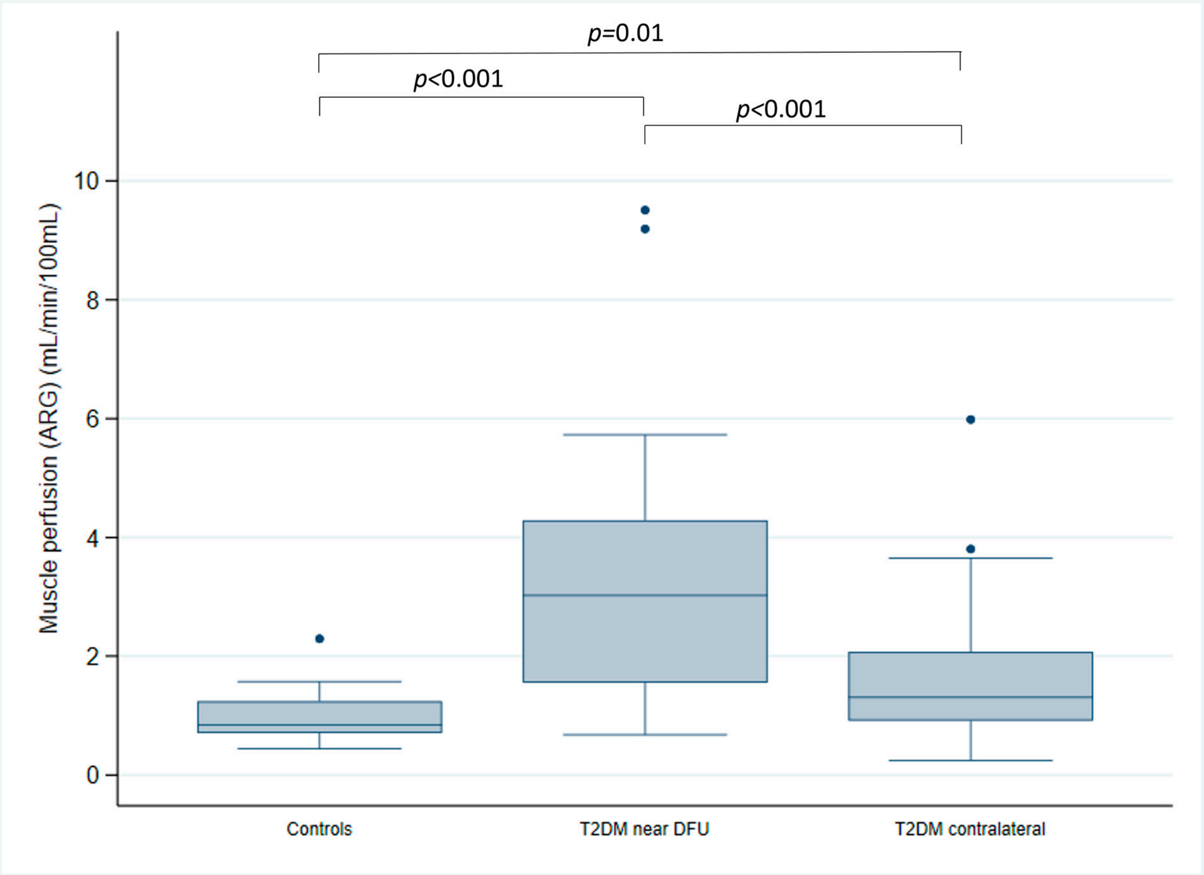

Figure S1: Comparison of muscle perfusion in controls (FHB muscle) and T2DM patients in the muscle closest to the DFU and the same muscle in the contralateral foot.

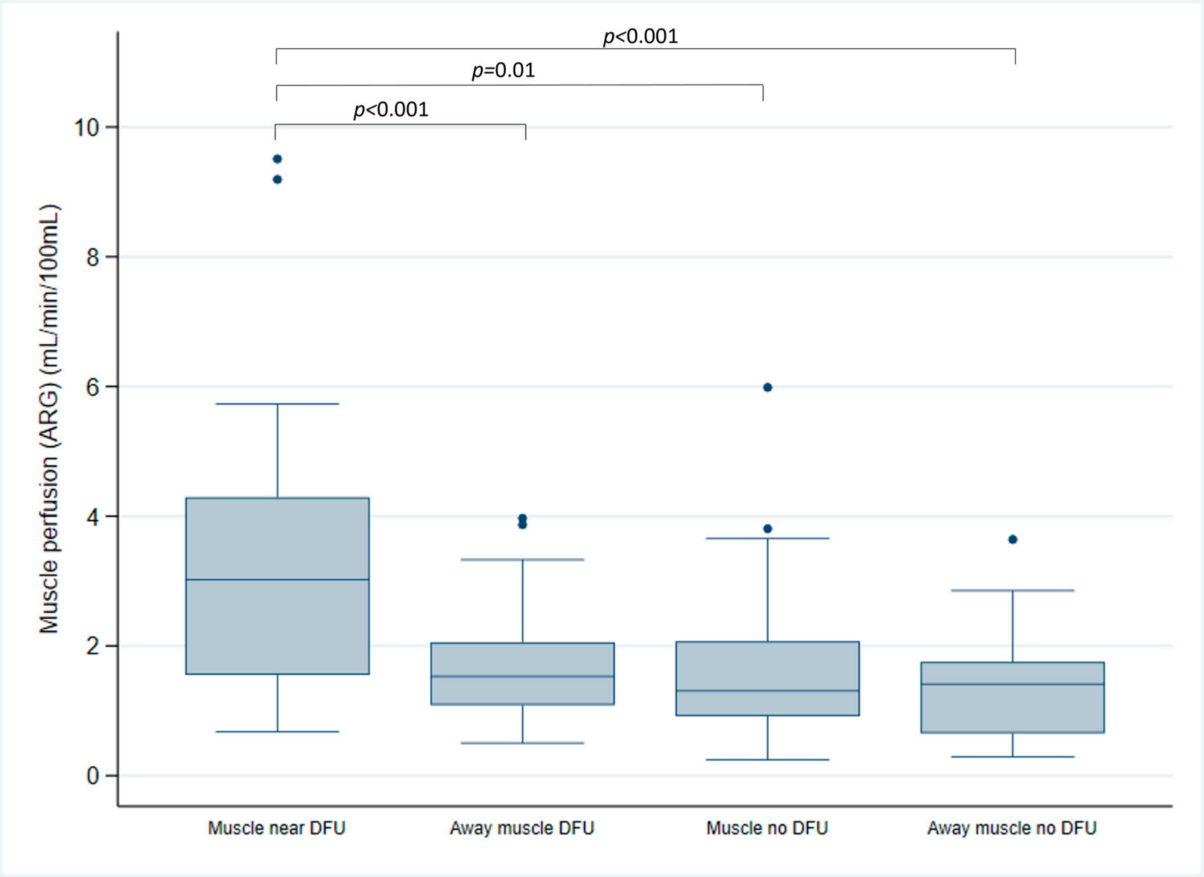

Figure S2: Comparison of skeletal muscle perfusion in the feet of T2DM patients. The box plot illustrates perfusion in the ulcerated foot, specifically in the muscle closest to the DFU (Muscle near DFU), in comparison to: perfusion in a muscle away from the ulcer in the same foot (Away muscle DFU), perfusion in the same muscle in the non-ulcerated foot (Muscle no DFU), and perfusion in the same muscle away from the ulcer in the non-ulcerated foot (Away muscle no DFU). Significance levels are indicated for the observed significant differences between groups.
